# A study of the phosphorylation proteomic skin characteristics of Tan sheep during the newborn and er-mao stages

**DOI:** 10.1007/s11250-021-02899-6

**Published:** 2021-12-29

**Authors:** Yonghong Chen, Dongqian He, Yachao Li, Fang Luo, Meng Zhang, Junkui Wang, Liyao Chen, Jinzhong Tao

**Affiliations:** Agricultural College, Ningxia University, Yinchuan, 750021 China

**Keywords:** Tan sheep skin, Er-mao stage fur, Phosphorylation, Proteomics

## Abstract

In this experiment, in order to study the formation mechanism of the lamb fur of Tan sheep, skin samples were collected from Tan sheep at the newborn and er-mao stages. Then, the phosphorylated proteomes of the skin samples of Tan sheep at the two different stages were compared and analyzed using a TMT labeled quantitative phosphorylation proteomic technique. A total of 2806 phosphorylated proteins were identified, including 8184 phosphorylation sites. The results of this study’s quantitative analysis showed that when compared with the skin samples at the er-mao stage, the phosphorylation levels of 171 sites had been upregulated in the skin samples at newborn stage. Meanwhile, 125 sites had been downregulated at the same stage. As shown by the results of the functional enrichment analysis of the differentially phosphorylated proteins, they had been mainly enriched in the cysteine and methionine metabolism. In addition, the phosphorylation levels of KAP4.7 and KAP13.1 had also varied during the different skin stages. These results indicated that the cysteine metabolism pathways, as well as the phosphorylation modifications of the keratin associated proteins in the skin, played important roles in the formation of the er-mao stage fur of the Tan sheep. Therefore, the findings of this study provided a new angle for interpreting the formation mechanism of er-mao stage fur properties.

**Supplementary Information:**

The online version contains supplementary material available at 10.1007/s11250-021-02899-6.

## Introduction

Tan sheep are an excellent breed of sheep used for fur in Ningxia. At the er-mao stage (approximately 35 days after birth), the wool is between 8 to 9 cm in length, characterized by wavy, white, and beautiful spikes. In addition, the fur is light and warm and obviously the top grade of fur (Tao et al. 2017; Kang et al. 2013). However, after 35 days, the hair plaits tend to become loose with non-compact tips, and the excellent properties of the er-mao fur gradually disappear. In recent years, some researchers have examined Tan sheep skin through proteomics analysis from the aspect of protein content in order to explore the formation mechanism of the specific fur properties of Tan sheep. The results of the aforementioned studies revealed that the differential expressions of the 14–3-3 protein σ subtype, trichohyalin (TCHH), KAP6, and KAP11.1 may be related to the formation of the wool fiber diameters, crimps, and specific flower form pattern of the Tan sheep (Yang et al. 2017; Kang et al. 2013). The existing relevant research enables us to have a certain understanding regarding the formation of specific fur properties of Tan sheep. However, the formation mechanism of the unique spike-type fur of Tan sheep has not yet been completely explained.

The phosphorylation of proteins is a reversible form of post-translational modification which is known to widely exist in organisms. It regulates such biological processes as cell signaling, gene expressions, molecular recognition, and metabolism (Zhai et al. 2008). As early as in 1996, some researchers proposed that phosphorylation played an important role in the assembly process of keratin intermediate filament (Ku et al. 1996). Subsequently, some studies found serine and tyrosine phosphorylation in II keratins (Herbert et al.^1997^). According to recent studies, phosphorylation may affect the development of animal hair follicles through TGF-β signaling pathway, as well as changing the development cycle of hair follicles, leading to changes in hair growth and hair quality (Foitzik et al. 2000). It has been determined that LRRK2 kinase specifically phosphorylates multiple Rab proteins. The phosphorylated Rab proteins combine with the primary cilia regulatory RILPL1/RILPL2, thereby affecting the formation of cilia (Steger et al. 2016).

In this study phosphorylation proteomics were applied to analyze the different phosphorylation proteins in the Tan sheep skin samples during the new born and er-mao stages. This study was to reveal the formation mechanism of the unique spike-type fur of Tan sheep.

## Materials and methods

### Sample collection

In the present study, three skin samples of winter lambs were collected at the newborn (1 to 2 days) and er-mao (35 days) stages, respectively, in a Ningxia Tan sheep breeding farm. The collected sample sites were 1 cm^2^ at the posterior edge of the scapula. The samples were rinsed with saline, immediately placed in a liquid nitrogen tank, and transported to laboratory facilities for storage at − 80 °C.

### Protein extraction and trypsin digestion

The obtained samples were removed from the − 80 °C storage conditions, and an appropriate amount of skin samples were placed into a pre-cooled mortar with liquid nitrogen and ground into powder, then received a 4 × volume lysis buffer (8 M urea, 1% protease inhibitor, 1% dephosphorylase inhibitor) for ultrasonic pyrolysis. Then, the samples were centrifuged under 12,000 g at 4 °C for 10 min in order to remove the remaining debris. The supernatant was transferred to a new centrifuge tube, and the protein concentration was determined using a BCA kit (see Supporting Information Table S1 for details of required materials and reagents). At that point in the experiment, dithiositol was added to the protein solution in order to achieve a final concentration of 5 mM, which was then reduced at 56 °C for 30 min. Then, iodoacetamide was added to achieve a final concentration of 11 mM, and the samples were incubated under dark conditions at room temperature for 15 min. Finally, the urea concentrations of the samples were diluted to less than 2 M. Trypsin was added at a mass ratio of 1:50 (trypsin:protein) for enzymolysis and the samples were stored overnight at 37 °C. After that, trypsin was added at a mass ratio of 1:100 (trypsin:protein) for a continued enzymolysis of four hours.

### TMT labeling and HPLC fractionation

The digested peptides were demineralized with Strata X C18 (Phenomenex) and then freeze-dried in a vacuum. The peptides were dissolved in 0.5 M TEAB and labeled according to the instructions of the TMT kit. This operation could be simply described as follows: (1) the labeled reagent was thawed, dissolved in acetonitrile, mixed with the peptides, and incubated at room temperature for two hours; (2) the labeled peptides were mixed, demineralized, and freeze-dried in a vacuum (see Supporting Information Table S2 for details of the sample labeling); (3) the peptides were fractionation by high pH reverse HPLC on Thermo Betasil C18 (5 μm particle size, 10 mm inner diameter, 250 mm length) as follows: 60 components were separated in 60 min with a peptide grading gradient of 8% to 32% acetonitrile and a pH of 9.0; (4) the peptides were combined into eight components, and the combined components were freeze-dried in a vacuum for subsequent operational processes.

### Enrichment of the phosphorylated peptides by IMAC

The present study, the peptides were dissolved in an enriched buffer solution (50% acetonitrile/6% trifluoroacetic acid), and the supernatant was transferred to pre-washed IMAC material. The samples were placed on a rotating shaker for incubation with gentle shaking. Following the incubation, buffer solutions of 50% acetonitrile/6% trifluoroacetic acid and 30% acetonitrile/0.1% trifluoroacetic acid were applied three times successively. Finally, the phosphopeptide was eluted with 10% ammonia water. The eluent was collected and drained using a vacuum freezing method. Following the draining process, it was demineralized according to the instructions provided by C18 ZipTips, and then drained by vacuum freezing for the subsequent liquid-mass analysis processes.

### Analyses using liquid chromatography-tandem mass spectrometry

Liquid chromatography-Tandem mass spectrometry(LC–MS, QE, plus, Thermo Scientific) was used, the peptides were dissolved in liquid chromatography mobile phase A (0.1% (v/v) formic acid solution) and then separated using an EASY-nLC 1000 ultra-high performance liquid phase system(Thermo Scientific). The mobile phase A was an aqueous solution containing 0.1% formic acid and 2% acetonitrile, and mobile phase B was an aqueous solution containing 0.1% formic acid and 90% acetonitrile. The liquid phase gradient settings were as follows: 0 to 38 min: 4% to 25% mobile phase B; 38 to 52 min: 25% to 6% mobile phase B; 52 to 56 min: 36% to 85% mobile phase B; and 56 to 60 min: 85% mobile phase B. The flow rate was maintained at 300 nL/min.

The peptides were separated using the ultra-high performance liquid phase system and injected into an NSI ion source for ionization. Then, the samples were analyzed using a Q ExactiveTM Plus (Thermo Scientific) mass spectrometry method. The ion source voltage was set at 2.0 kV, and the peptide parent ions and their secondary fragments were detected and analyzed with a high resolution Orbitrap. The scanning range of the primary mass spectrometry was set at between 350 and 1800 m/*z*, and the scanning resolution was set as 70,000. The scanning range of the secondary mass spectrometry was fixed at 100 m/*z*, and the resolution of secondary scanning was set at 17,500. A data-dependent acquisition (DDA) procedure was used in this study’s data collection mode. For example, the first 20 peptide parent ions with the highest signal intensity were selected to successively enter the high energy collision dissociation (HCD) collision pool after the primary scanning was completed. Then, 28% of the fragmentation energy was used for the fragmentation. The secondary mass spectrometry analysis was also carried out in the same way. In order to improve the effective utilization of mass spectrometry and avoid repeated scanning of the parent ions, the automatic gain control (AGC) was set as 5E4; signal threshold was 5000 ions/s; maximum injection time was 200 ms; and the dynamic exclusion time of the tandem mass spectrometry scanning was set as 15 s.

### Database search

Maxquant (v1.5.2.8) software was used to retrieve the secondary mass spectrometry data. The retrieval parameter settings were as follows: (1) the database Uniprot Ovis Aries (23,111 sequences) was added to the inverse library in order to calculate the false discovery rate (FDR) caused by random matching; (2) a common contamination library was added to eliminate the influences of contaminated proteins in the identification results; (3) Trypsin/P was specified as the cleavage enzyme allowing up to two missing cleavages; (4) the minimum length parameter of the peptide was set as 7 amino acid residues, and the maximum number of modifications per peptide is set as 5; (5) the tolerance rates for mass errors of the primary parent ions in the first search and main search were set as 20 ppm and 5 ppm, respectively, and the tolerance for mass errors of the secondary fragment ions was set as 0.02 Da; (6) cysteine alkylation was set as a fixed modification, and the variable modifications were the oxidation of methionine, acetylation of the N-terminal of the protein, and phosphorylation of serine, threonine, and tyrosine; (7) the quantitative method was set to TMT-6plex, and the FDR for the protein and PSM identification was set as 1%.

### Bioinformatics analysis

#### Analysis of the protein functional enrichment

The GO annotation (UniProt-GOA, www.http://www.ebi.ac.uk/GOA/) of the proteins was divided into three categories as follows: Biological processes; cellular components; and molecular functions. For each category, a two-tailed Fisher’s exact test was employed for the purpose of testing the enrichment of the differentially modified proteins against all the identified proteins. The GO with corrected *p*-values < 0.05 were considered to be significant.

The KEGG database (KAAS, v.2.0 http://www.genome.jp/kaas-bin/kaas_main; KEGG Mapper, V2.5 http://www.kegg.jp/kegg/mapper.html) was used for pathway enrichment analysis and a two-tailed Fisher’s exact test was used to test the differentially modified proteins. Then, based on the identified proteins, the pathway enrichment test results with *p* < 0.05 were considered to be significant. Finally, these pathways were classified according to the hierarchical classification method of pathways available on the KEGG website.

The InterPro database was examined and a two-tailed Fisher’s exact test was employed to test the enrichment of the differentially modified proteins against all of the identified proteins. The protein domains with corrected *p*-values < 0.05 were considered to be significant.

#### Motif analysis of the phosphorylated proteins

Motif-x software was adopted in this study to analyze the motif characteristics of the phosphorylation sites. The identified peptide sequences composed of six amino acids in the upstream and downstream of the phosphorylation sites were chosen as the analysis objects. When the number of peptides in a specific feature sequence was greater than 20 with *p* < 0.000001, these were statistically tested, and the feature sequence was considered to be a motif of the modified peptide.

## Results and analysis

### Quality control analysis of the phosphorylated proteome

**Fig. 1:**
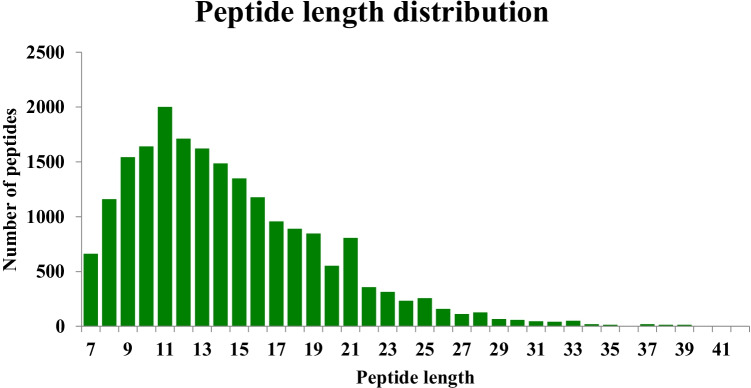
Quality control test results of the length distribution of the peptides identified by mass spectrometry

As shown in Fig. 1, the majority of the peptide lengths were distributed between 8 and 20 amino acid residues, which was consistent with the rule of trypsin digesting. This indicated that sample had reached the standard.

**Fig. 2:**
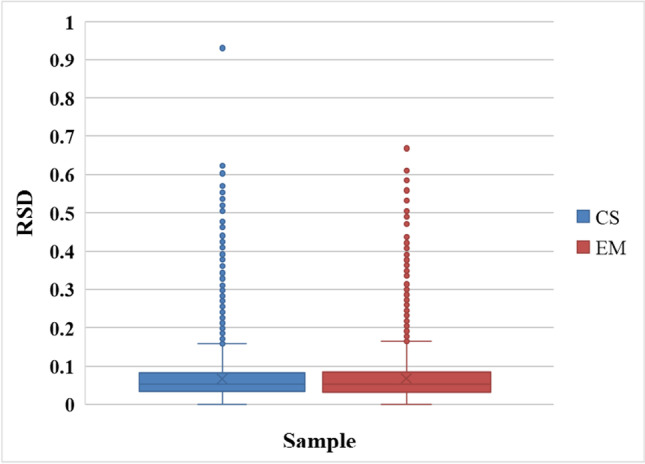
Sample repeatability test. CS, newborn; EM, Er-mao

As detailed in Fig. 2, a box-plot was drawn with the relative standard deviation (RSD) calculated using the repeated experimental values of each group of samples. The repeatability of the tested samples was found to be strong and statistically consistent.

### Quantitative results of the modified sites and proteins

**Fig. 3:**
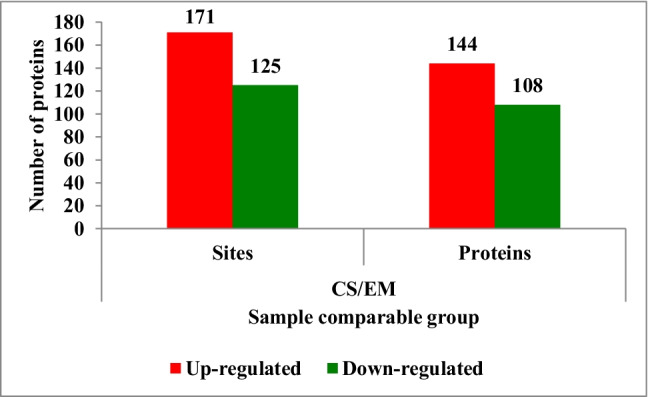
Statistical information of the differential expressions of phosphorylation

**Fig. 4:**
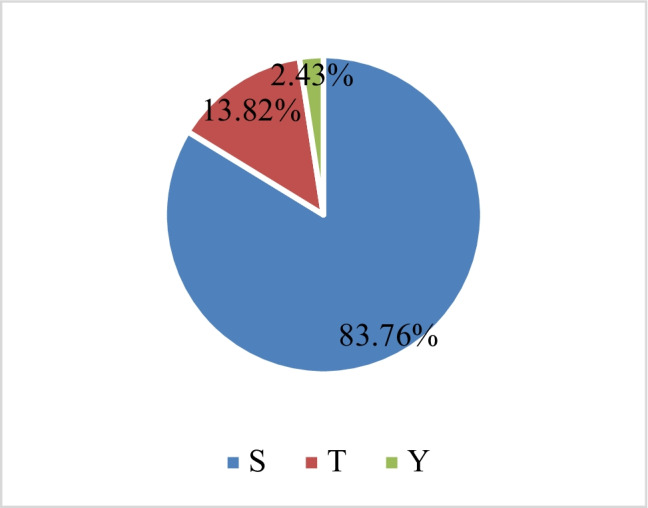
Distribution of the Ser/Thr/Tyr phosphorylation sites

In this study, a total of 8184 phosphorylation sites were identified on 2806 proteins, of which 6773 sites on 2663 proteins displayed quantitative information (Table 1). In order to ensure the reliability of the testing results, the identification data were filtered with a standard of localization probability > 0.75. Phosphorylation sites of the proteins identified in the newborn group were compared with those in the er-mao group (CS/EM). The protein phosphorylation sites with ratios > 1.3 and *p* < 0.05 were considered to have been significantly upregulated. Meanwhile, those with ratios < 1/1.3 and *p* < 0.05 were considered to be significantly downregulated (as illustrated in Fig. 3). A total of 171 significantly upregulated phosphorylation sites on 144 proteins, and 125 significantly downregulated phosphorylation sites on 108 proteins were detected. The modification information of some of the proteins in the phosphorylation sites are listed in Table 2. Among the identified 8184 phosphorylation sites, the phosphorylation sites of serine, threonine, and tyrosine accounted for 83.76%, 13.82%, and 2.43% of the total, respectively (Fig. 4).

**Table 1:** Statistical information on the identification and quantification of protein phosphorylation modifications

	Identified (localization probability > 0.75)	Quantifiable (localization probability > 0.75)
Sites	8184 (5576)	6773 (5417)
Proteins	2806 (2510)	2663 (2455)

The sites with a localization probability > 0.75 are indicated in brackets, along with the corresponding proteins.

**Table 2:** Partial differential modification sites and corresponding proteins

Protein accession	Position	CS/EM ratio	Regulated type	Amino acid	Protein description
W5Q6P5	454	2.21	Up	S	Hair keratin type II intermediate filament
W5P696	12	1.808	Up	S	Protein phosphatase 1 regulatory inhibitor subunit 14A
W5PTV4	106	1.715	Up	S	Phosphodiesterase
W5PBG5	1310	1.498	Up	S	Inhibitor of Bruton tyrosine kinase
W5Q2K6	140	1.476	Up	S	Keratin-associated protein 4–7 OS = Homo sapiens GN = KRTAP4-7 PE = 1 SV = 2
W5P1D0	129	1.376	Up	S	Cyclin-dependent kinase 18 OS = Pongo abelii GN = CDK18 PE = 2 SV = 2
W5NXL1	444	1.363	Up	S	Calcium/calmodulin dependent serine protein kinase
W5NQR0	840	1.355	Up	T	Serine/arginine repetitive matrix 2
W5P5V3	73	1.352	Up	S	NADH:ubiquinone oxidoreductase subunit B7
W5Q478	259	1.347	Up	S	Mitogen-activated protein kinase 8 interacting protein 3
W5NRV6	98	0.477	Down	S	Keratin-associated protein 13–1 OS = Homo sapiens GN = KRTAP13-1 PE = 2 SV = 2
W5PIJ2	197	0.538	Down	S	Adenosylhomocysteinase
W5PXC8	484	0.572	Down	Y	Serpin family F member 2
W5Q1L5	394	0.591	Down	Y	Non-specific serine/threonine protein kinase
W5Q5H8	448	0.613	Down	T	Fibrinogen alpha chain
W5PNA9	29	0.649	Down	S	Cystathionine beta-synthase
W5Q2D7	821	0.688	Down	Y	Protein tyrosine phosphatase, receptor type C
W5QF57	351	0.689	Down	S	Tyrosine-protein phosphatase
W5Q6L8	16	0.695	Down	S	Keratin 14
W5QCC0	734	0.709	Down	T	Ral GTPase activating protein non-catalytic beta subunit

### Bioinformatics analysis

#### Functional annotation and enrichment analysis of the proteins

**Fig. 5:**
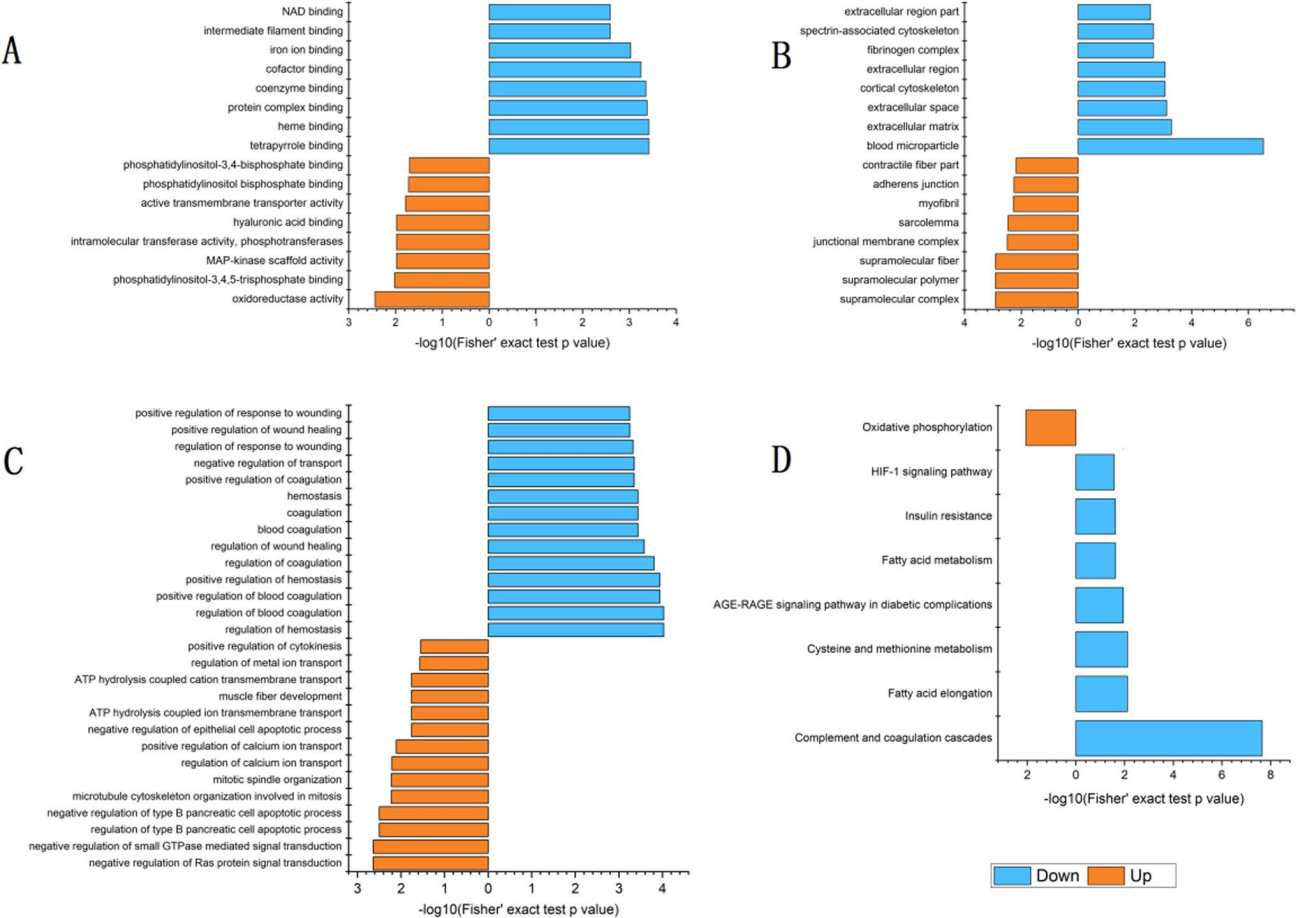
**A** Molecular function enrichment of GO of differentially modified proteins; **B** cellular component enrichment of GO of differentially modified proteins; **C** biological process enrichment of GO of differentially modified proteins; **D** KEGG pathway enrichment of differentially modified proteins. *Note*: In the figure, the horizontal axis value was the negative log conversion of the significant *p*-values (*p* < 0.05)

GO enrichment analysis result that, among the proteins corresponding to the upregulated phosphorylation sites, the phosphorylated proteins which were related to the cellular components were mainly enriched in the supramolecular complexes, supramolecular polymers, supramolecular fibers, junctional membrane complexes, and sarcolemma. In addition, it was observed that the phosphorylated proteins which were enriched in molecular function were mainly involved in oxidoreductase activities and phosphatidylinositol-3,4,5-trisphosphate binding. Furthermore, many of the phosphorylated proteins were found to be enriched in various biological processes, such as the negative regulation of Ras protein signal transduction, negative regulation of small GTPase mediated signal transduction, regulation of type B pancreatic cell apoptotic processes, negative regulation of type B pancreatic cell apoptotic processes, and the microtubule cytoskeleton organization involved in mitosis. It was also found that the downregulated proteins were enriched in the molecular functions of tetrapyrrole binding, heme binding, protein complex binding, and coenzyme binding. In addition, they were enriched in such biological processes as the regulation of hemostasis, regulation of blood coagulation, positive regulation of blood coagulation, and positive regulation of hemostasis, as well as being mainly enriched in such cellular components as blood microparticles, extracellular matrixes, and extracellular spaces (Fig. 5)

It was found that the upregulated phosphorylated proteins were mainly concentrated in the oxidative phosphorylation pathway. However, the downregulated proteins were mainly enriched in fatty acid elongation, cysteine and methionine metabolism, fatty acid metabolism, and other pathways, as detailed in Fig. 5.

#### Motif analysis of the protein modification

**Fig. 6:**
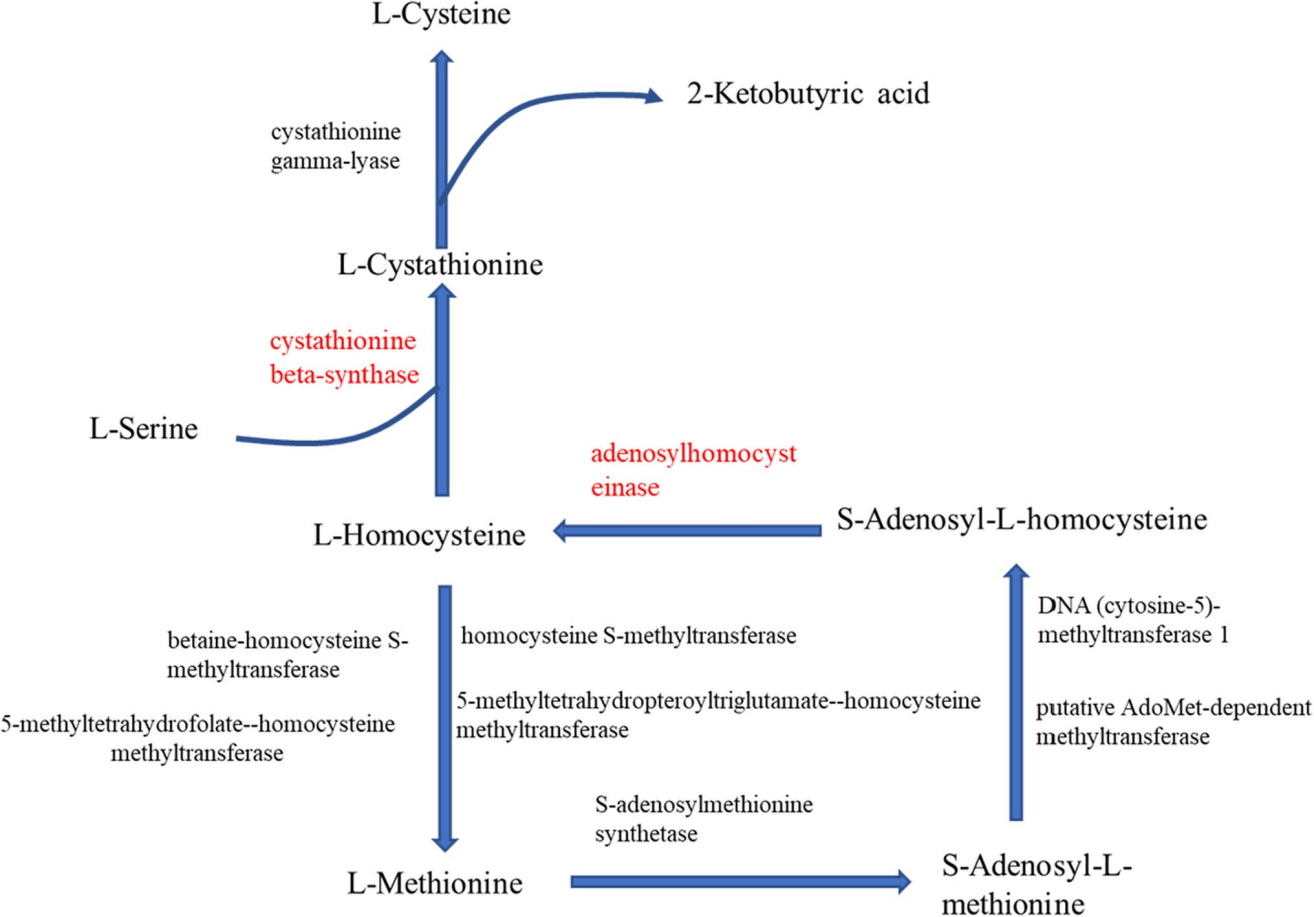
Schematic diagram of cysteine and methionine metabolism pathways based on the KEGG pathway enrichment. *Note*: In the figure, the red letters represent two proteins with significantly reduced phosphorylation levels; the arrows indicate the directions of the material metabolism

**Fig. 7:**
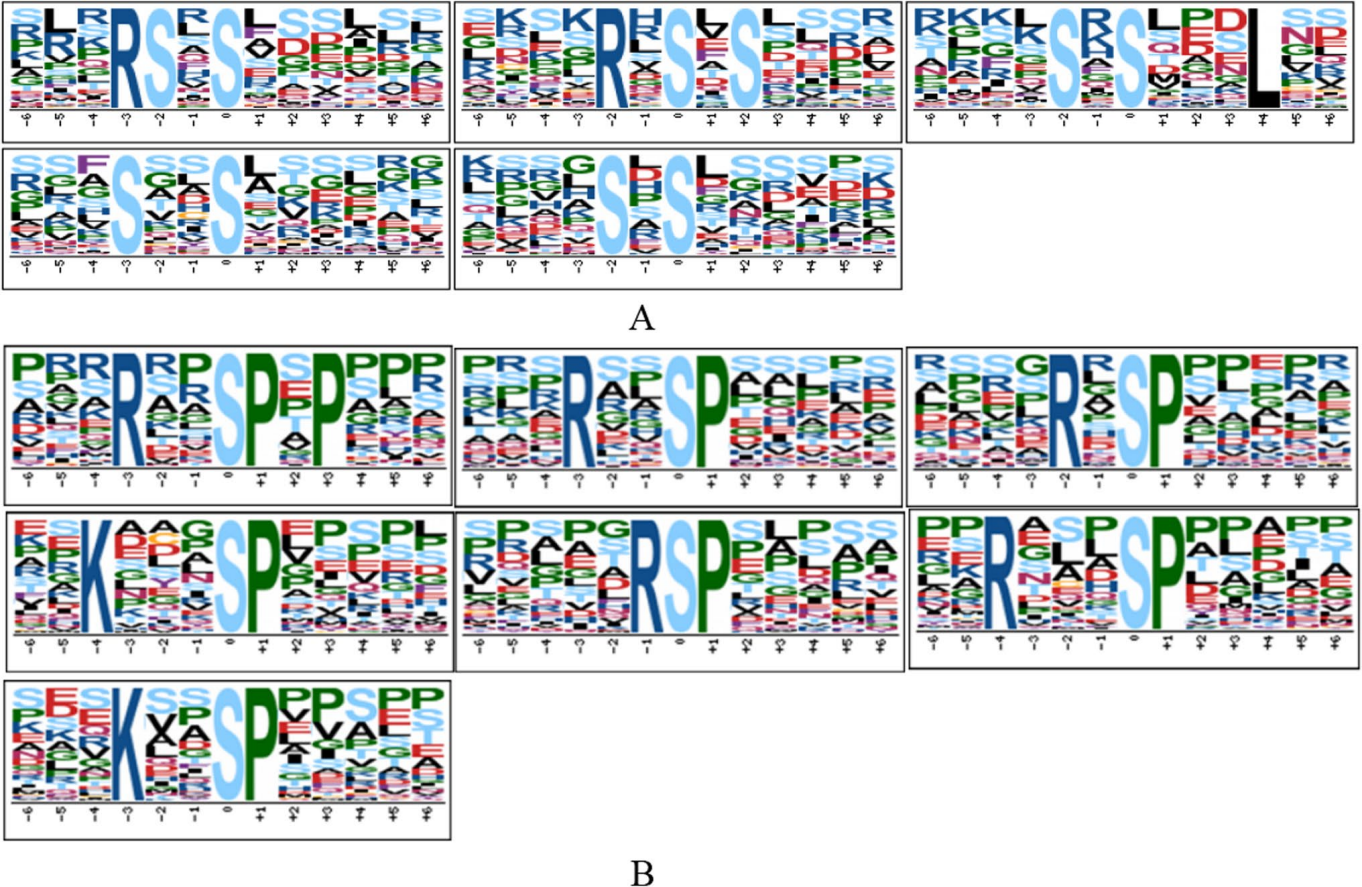
**A** Indicates the motifs with dual phosphorylation sites, and **B** indicates the motifs of both the B and P types

A total of 35 serine phosphorylation sequences and three threonine phosphorylation sequences were detected in motif analysis results, as detailed in Supporting Information Table S3. Among the 35 serine phosphorylation motifs, five motifs with two serine phosphorylation sites were found (Fig. 6A). Then, in accordance with the specificity of the substrate sequence of serine and threonine protein kinase determined in earlier studies, the serine and threonine phosphorylation site motifs could be divided into four categories (Villen et al. 2007; Amanchy et al. 2007), as follows: Pro-directed: P; acidic: A; basic: B; and other kinases: O. Then, on the basis of the aforementioned rule, seven motifs were determined to belong to both the B and P types (Fig. 6B). In addition, it was found that the statistics of the motifs of type P accounted for 39.59%; type A accounted for 8.16%; type B comprised 26.14%; type O made up 11.17%; and the motifs belonging to both B and P types accounted for 14.93%, as detailed in Fig. 7A.

## Discussion

### Motif analysis of the phosphorylated protein in the skin of Tan sheep

The amino acid sequences adjacent to serine, threonine, or tyrosine usually determine the specificity of the kinase. Therefore, the identifications of amino acid enrichment around phosphorylation products are of important reference value for the identification of kinase activities(Ren et al. 2018). In this study, the motifs of P type accounted for the majority (39.59%). B type motif (26.14%) was followed, then those with both B and P types (14.93%). Among the motifs corresponding to 296 differential modification sites, 89, 94, and 25, respectively, were matched to the above-mentioned motif types (Fig. 7B), and accounted for 70.2% of the total. The most prominent feature of all the P type motifs was the presence of a proline residue at the + 1 position of S/T. That is to say, all of the motifs of the P type, as well as those of both B and P types, could be combined into “……SP…..”. According to previous relevant studies, glycogen synthase kinase 3, cyclin-dependent Kinase 5 (CDK5), and mitogen-activated protein kinase (MAPK), mainly act on Pro-directed phosphorylation sites(Li et al. 2006; Olsen et al. 2010). The B type motifs of the differentially modified protein sites mainly included the following: “…RR.S……”, “…RS.S……”, “…R..S……”, “….R.S……”, and “…K..S……”. As shown by existing study results, they had been phosphorylated mainly by protein kinase A and protein kinase C (Ren et al. 2018; Olsen et al. 2010; Perason and Kemp 1991), which indicated that the same phosphorylation site of the same protein was regulated by one or more kinases. In addition, the motifs of both the P and B types have been previously reported (Kwon et al. 2016). Therefore, it was concluded in this study that glycogen synthetase kinase 3, cyclin-dependent kinase 5 (CDK5), mitogen-activated protein kinase (MAPK), protein kinase A, and protein kinase C may play important roles in regulating phosphorylation reactions during the development of the wool fibers in Tan sheep.

### Effects of the phosphorylation of KAP on the properties of Tan sheep fur during the er-mao stage

KAP4.7 is a component of wool and also a member of the ultra-high sulfur protein family (Plowman et al. 2018a), and the protein is only expressed in the paracortex of hair follicles(Plowman et al. 2018b). The previous studies have shown that the expressions of KAP4.7 in white Merino sheep are higher than that in black Merino sheep, which is consistent with the results of larger wool fiber diameters and smaller crimping in black Merino sheep. The reason for this mainly lies in the decreases in paracortical protein proportions (Almeida et al. 2014; Plowman et al. 2019). The asymmetric expressions of KAPs hair follicles on both sides of the orthocortex and paracortex have been considered as one of the factors causing the crimps in wool fiber (Caldwell et al. 2005; Yu et al. 2009). In this study, it was found that the phosphorylation levels of KAP4.7 at Ser140 during the newborn stage of the Tan sheep were significantly higher than those during the er-mao stage. Generally speaking, there were approximately 5 crimp numbers in the wool fiber of the Tan sheep at the newborn stage, and 6 to 9 crimp numbers at the er-mao stage and wools were straight near skin. The phosphorylation of the proteins tends to affect the spatial conformation of the proteins and their mutual interactions (Mijakovic et al. 2016). Meanwhile, the spatial structures of the KIFs and their chemical binding with the KAPs in the matrix determine the physical properties of the fibers to a great extent (Powell and Rogers 1997). Therefore, it has been speculated that the low-level phosphorylation of KAP4.7 at the Ser140 site interact with keratin intermediate filaments, and other KAPs, and then affect the formations of crimps in the fur during the er-mao stage of the Tan sheep.

KAP13.1 is a wool structural protein and also a member of a high-sulfur protein family, and expressed in the cortex layer of hair follicles (Plowman et al. 2018b). It is one of the first expressed protein in sheep hair follicles (Plowman et al. 2015), but the second expressed protein in human hair follicles (Rogers et al. 2001). ITRAQ proteomics were used to identify significant increases in KAP13.1 expressions and significant decreases of fiber diameters in Merino sheep undergoing restricted feeding conditions (Almeida et al. 2014). 25.7 mol% serine was found in the KAP13.1; then, it is prone to phosphorylation (Plowman et al. 2018a; Gong et al. 2011). This study found that the phosphorylation levels of the KAP13.1 at Ser98 during the er-mao stage were significantly higher than that observed during the newborn stage. It was speculated that the high phosphorylation of KAP13.1 at Ser98 during the er-mao stage may potentially affect the internal structures of the wool fibers by affecting the binding of the KAP13.1 and keratin, thereby impacting the formation of Chuanzihua type wool fibers. Hovever, so far, mechanism of KAP13.1 effect wool crimp is unclear.

### Effects of phosphorylation in the metabolism of methionine and cysteine on the fur properties of Tan sheep during the er-mao stage

Methionine is a limited amino acid which can be decomposed into cysteine through the decomposition of sulfur or amino groups (Reis 1982). The content levels of cysteine play important roles in the synthesis of wool fiber. Many studies have shown that the content of sulfur-containing amino acids will affect the quality of the wool fiber, such as the fineness, length, cashmere yield and other physical properties (Gillespie and Reis 1966; Reis and Sahlu 1994). In addition, with the increases in sulfur content, the diameters of the wool fibers will become smaller. Furthermore, for Merino sheep, it has been determined from relevant research results that the fibers with high curvature characteristics contained 20% more cysteine than those with low curvatures (Campbell et al. 1972). Also, approximately 60% of the cysteine in a sheep’s diet is converted to wool (Hogan et al. 1979), and approximately 97.5% of the cysteine in the wool forms disulfide bonds (Fraser et al. 1988), which subsequently results in bridges between the KAPs and keratin intermediate filaments.

**Fig. 8:**
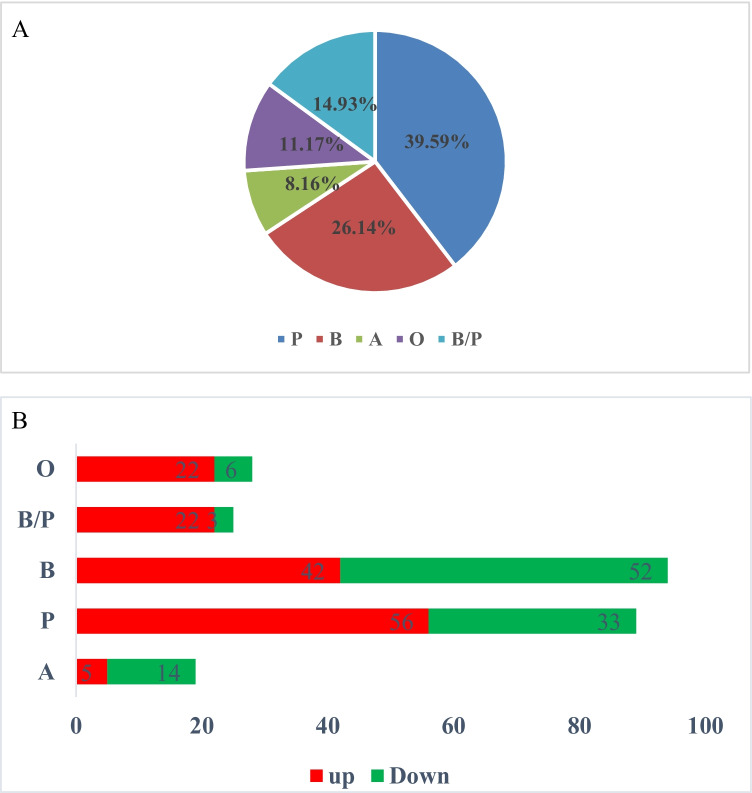
**A** Indicates the protein distributions of the phosphorylated motifs of the different proteins, and **B** indicates the number distribution of the differential protein modification sites matched to the different motif types

In the current study, the results of the KEGG pathway enrichment analyses revealed significant differences in the phosphorylation levels of cystathionine-β-synthase and adenosyl homocysteinase related to methionine and cysteine metabolism. It was observed that the phosphorylation of cystathionine-β-synthase at the Ser29 site was significantly downregulated during the newborn stage. The KEGG pathway analysis results showed that the protein mainly played a role in the process of L-homocysteine conversion to L-cystathionine (Fig. 8). It acted as the only enzyme to promote the synthesis of L-cysteine. L-cystathionine is the substrate for the synthesis of L-cysteine, and L-cysteine can be produced by the action of cystathionine-γ-lyase. It was observed in this study that the phosphorylation of adenosyl homocysteinase at the Ser197 site was significantly downregulated during the newborn stage. The results of the KEGG pathway analysis showed that protein mainly played a role in the process of S-adenosyl-L-homocysteine conversion to L-homocysteine. It had acted as the only enzyme to promote the synthesis of L-homocysteine. L-homocysteine synthesizes L-cystathionine by cystathionine-β-synthase, and converts to L-methionine by various enzymes, and S-adenosyl-L-methionine synthesizes from L-methionine. As mentioned above, the S-adenosyl-L-homocysteine was converted into L-homocysteine.

It is well known that wool fiber is composed of α-keratin assembled into keratin intermediate filaments, embedded into matrix proteins, and then cross-linked with three major types of KAPs matrix protein through disulfide bonds, with the cross-linking effects of the disulfide bonds mainly formed by cysteine (Plowman et al. 2018a; Gong et al. 2019). It was found that the wool fibers of the Tan sheep during the er-mao stage featured longer wool fibers, with increased amounts of crimps and finer wool growth. Therefore, the different phosphorylation levels of cystathionine-β-synthase Ser29 and adenosyl homocysteinase at the Ser197 site may have potentially affected the synthesis of L-cysteine during the er-mao stage of Tan sheep through the activities of those two enzymes.

## Supplementary Information

Below is the link to the electronic supplementary material.

Supplementary file1 (DOCX 204 KB)

## Data Availability

I promise that all data in this paper are true and reliable, and all data have been verified by Chinese government authorities (third party testing data). This manuscript is original and has not been submitted elsewhere for publication.
