# Supplementary material for: A study of the phosphorylation proteomic skin characteristics of Tan sheep during the newborn and er-mao stages
Source: Trop Anim Health Prod. 2021 Dec 29;54(1):30. doi: 10.1007/s11250-021-02899-6 (PMC8714624; doi:10.1007/s11250-021-02899-6)

**Supplementary material 1**

Sample Labeling information

| samples | labeling | labeling efficiency |
| --- | --- | --- |
| CS-1 | 126 | 99.10% |
| CS-2 | 127 |  |
| CS-3 | 128 |  |
| EM-1 | 129 |  |
| EM-2 | 130 |  |
| EM-3 | 131 |  |

Note: Detection of labeling efficiency ① Detection of labeling efficiency: 5μ L of peptide from each sample was drained, 0.1% TFA was added and dissolved until the concentration of peptide was 0.1μg/μ L, 10% TFA (about 2μ L) was adjusted to pH less than 3, and 50μ L (5μg) was mixed in equal amounts. The labeling efficiency was checked by Stage X demineralization and mass spectrometry.

② Demineralization: The labeling efficiency is greater than 97%, the sample is drained, 0.1% TFA is added to dissolve, 10% TFA (about 13 μ L) is adjusted to pH less than 3, the same amount of mixing, strata X demineralization;

**Supplementary material 2**

The fine location of the modification sites of the KAP4.7 and KAP13.1(Protein accession, W5Q2K6, W5NRV6)


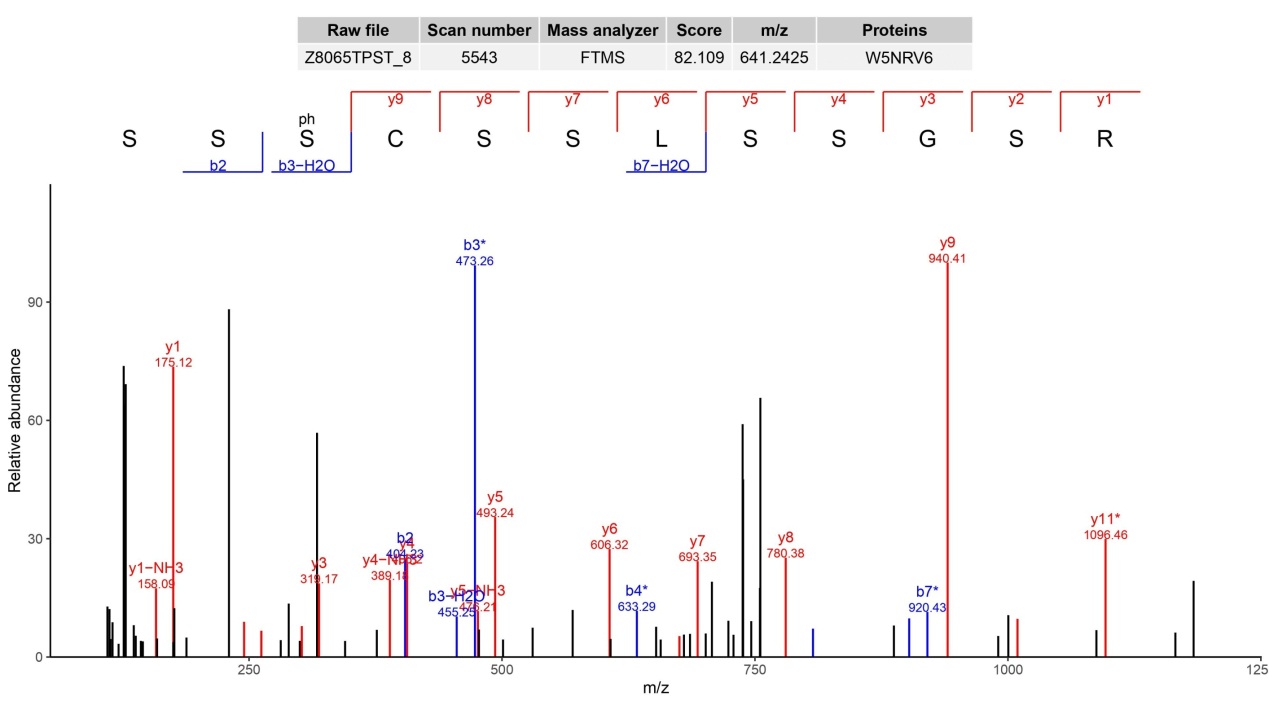


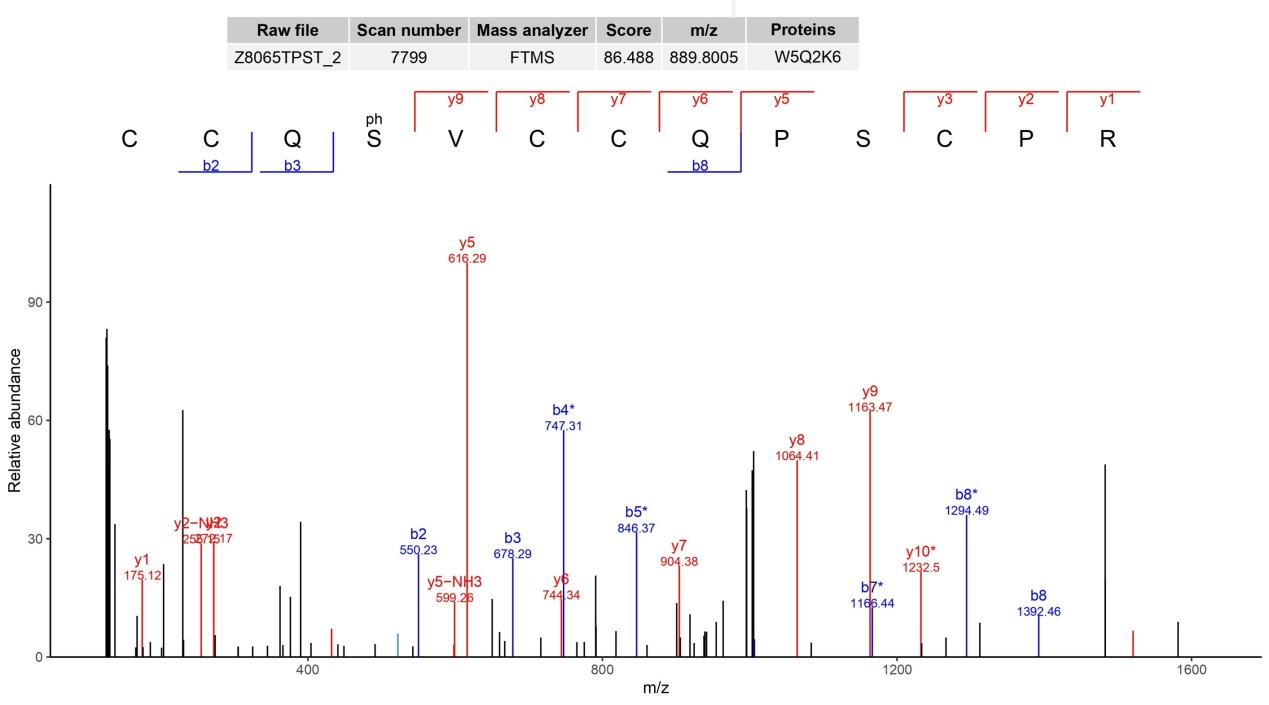

Supplement: Supplementary file 1 — Supplementary file1 (DOCX 204 KB) [file 11250_2021_2899_MOESM1_ESM.docx]
